# The Relationship Between Periodontal Disease (Pd) and Cardiovascular Disease (Cvd).

**DOI:** 10.4084/MJHID.2010.030

**Published:** 2010-10-01

**Authors:** Maurizio Trevisan, Joan Dorn

**Affiliations:** 1 Health Sciences System of the Nevada System of Higher Education, Las Vegas, Nevada USA; 2 School of Public Health and Health Professions, State University of New York at Buffalo, Buffalo New York, USA

## Abstract

The recent focus on the potential link between periodontal and cardiovascular disease (PD and CVD) is part of the larger renewed interest on the role of infection and inflammation in the etiology of atherosclerosis and its clinical manifestations. Periodontal Disease is an inflammatory process affecting the periodontium, the tissue that surrounds and supports the teeth. The process usually starts with an inflammatory process of the gum (gingivitis) but it may progress with an extensive involvement of the gum, as well as the periodontal ligament and the bone surrounding the teeth resulting in substantial bone loss. Periodontal disease is a common oral pathological condition in the adult age and represents the leading cause of tooth loss. PD prevalence increases with age and there are estimates that up to 49,000,000 Americans may suffer from some form of gum disease. The gingival plaque associated with PD is colonized by a number of gram-positive and gram-negative bacteria that have been shown to affect the initiation and development of PD and have been associated with the potential etiological role of PD in CVD and other chronic conditions. A potential etiological link between PD and CVD may have important public health implications as both the exposure (PD) and the outcomes (CVD) are highly prevalent in industrialized societies. In situations in which both the exposure and the outcome are highly prevalent even modest associations, like those observed in the studies reporting on the link between PD and CVD outcomes, may have relevance. There are not definite data on the effect of periodontal treatment on CVD clinical outcomes (either in primary or secondary prevention) however it should be pointed out that the limited (both in terms of numbers and study design) experimental evidence in humans suggests a possible beneficial effect of periodontal treatment of indices of functional and structural vascular health.

## Introduction:

The recent focus on the potential link between periodontal and cardiovascular disease (PD and CVD) is part of the larger renewed interest on the role of infection and inflammation in the etiology of atherosclerosis and its clinical manifestations. In this review, we will describe the potential mechanisms that have been identified and review the evidence from observational and intervention studies.

Periodontal Disease is an inflammatory process affecting the periodontium, the tissue that surrounds and supports the teeth. The process usually starts with an inflammatory process of the gum (gingivitis) but it may progress with an extensive involvement of the gum, as well as the periodontal ligament and the bone surrounding the teeth resulting in substantial bone loss. Periodontal disease is a common oral pathological condition in the adult age and represents the leading cause of tooth loss. PD prevalence increases with age and there are estimates that up to 49,000,000 Americans may suffer from some form of gum disease.

A hallmark of PD is the presence of bacteria in the gingival plaque (both supra- and sub-gingival) that characterizes the periodontal pathological process. The oral cavity is colonized by hundreds of bacteria, most of which have no clear pathological effects; however the gingival plaque associated with PD is colonized by a number of gram-positive and gram-negative bacteria that have been shown to affect the initiation and development of PD and have been associated with the potential etiological role of PD in CVD and other chronic conditions.

## Mechanisms:

A number of mechanisms have been hypothesized to explain the potential pathological role of periodontal disease in cardiovascular disease etiology. These include: a) potential direct mechanisms on the vessel wall and the atherosclerotic plaque and b) indirect mechanisms.

### Direct Mechanisms:

This hypothesis assumes that bacteria or their products access the vessel wall (endothelium) directly, through the blood stream, and affect either the formation of the plaque and/or its evolution. In support of this hypothesis a number of studies have shown evidence of presence of viable oral bacteria or their genetic material in atheromatous plaque samples from different vascular beds.^1^–^4^ However, not all the studies have confirmed these findings.^5^,^6^

### Indirect Mechanisms:

Indirect mechanisms through which PD can affect CVD include the potential effects on a number of classical CVD risk factors like total and LDL serum cholesterol, blood pressure, glucose metabolism and platelet aggregation. A number of observational studies have shown significant associations between PD and serum total cholesterol^7^–^9^ and PD and glucose metabolism,^10^,^11^ the observational association between PD and blood pressure has been less investigated;^12^ however a small randomized clinical trial has shown significant effects of periodontal therapy on both these risk factors.^13^ No consistent evidence has been generated supporting an effect of PD on either HDL or triglycerides. The mechanisms underlying these effects are not clear but may be related to the effect of PD on endothelial function or the inflammatory process. An additional mechanisms through which PD can affect CVD is through an effect on platelet aggregation.^14^

Substantial efforts have been dedicated to investigate the role of inflammation and its biomarkers in the link between PD and CVD.

As previously indicated hallmarks of PD are the presence of bacteria and the local inflammatory process.^15^ There are strong indications that the periodontal pocket can act as the port of entry in the organism of both bacteria and their products and inflammatory and pro-inflammatory mediators. The result, either through transient bacteremia or direct access to the blood stream of inflammatory mediators, is a generalized inflammatory process.

An additional mechanism that has been hypothesized to play a role in the link between PD and CVD is molecular mimicry.^16^ This hypothesis is based on the relevance of the immune response in atherogenesis. According to this hypothesis bacterial products and/or inflammatory mediators may induce the endothelium to produce host protective factors against the bacterial heat shock proteins (HSP). This immune response targeted against specific HSP antigens in plaques may exacerbate the established inflammation thereby promoting atherosclerotic plaque progression and stability.

## The exposure:

The measurements of Periodontal disease/health that have been utilized to date vary substantially, and include: general measurements of oral health, crude measurements of self-reported missing teeth, standardized measurements of gingival detachment (periodontal pockets), radiographic measurements of bone loss and more recently measurements of bacterial infection. The interpretation of the literature is complicated by the lack of an agreement on a clear definition of PD in the scientific community. In general there is consistency in findings across studies using different indices of PD and the studies that have used more detailed measurements of exposures have, in general, showed stronger association between PD and CVD outcomes.

## The outcomes:

Different outcomes have been investigated and include both clinical outcomes (i.e. Coronary Heart Disease, Myocardial Infarction, Acute Coronary Syndrome, and Stroke) as well as non-invasive measurements of subclinical atherosclerosis [i.e. carotid intima media thickness (IMT) and endothelial dysfunction].

In addition to these disease related outcomes a number of studies (both observational and intervention in nature) have investigated the relationship between PD and a wide array of cellular and plasma markers of inflammation (i.e. White blood cells, CRP, cytokines).

## The evidence

### Observational Studies:

The evidence to date relating PD to clinical outcomes is based solely on observational studies. These studies include clinical comparisons of selected samples, retrospective (case-control) and longitudinal epidemiological studies.

### Coronary Artery Disease:

A total of approximately sixty studies have focused on cardiac outcomes (i.e. CHD, MI or ACS) and provided quantitative assessment of risk. The majority but not all these studies show a significant association between PD and these clinical outcomes. Many of the studies presented multivariate adjusted estimates indicating that the relationship may be independent from the potential confounding effect of socio-economic factors, life style habits like smoking and other important covariates. This association has been consistently found in studies from different parts of the world and in both men and women. The evidence in women is much more limited but a study that focused on both sexes provided evidence for a potentially higher risk of MI in women compared to men.^17^ The increased risk of coronary events in individuals with PD has been shown both in subjects with and without pre-existing CVD.^18^–^22^

Several reviews have indicated an overall significant association between PD and coronary outcomes, two more recent systematic reviews indicated that relative risk estimates ranged from a 24% to a 34% increased risk and that these estimates were consistent across the various methods to ascertain both PD and coronary events.^23^–^24^

### Stroke:

The amount of articles reporting on the link between PD and cerebrovascular outcomes are more limited but, as for coronary outcomes, the overall evidence is in support of a positive association. The evidence appears to be stronger for studies with more detailed measures of PD and to be present for both fatal and non fatal events.^25^–^28^ A study that analyzed both hemorrhagic and non hemorrhagic stroke reported a significant association with the latter and not the former outcome, providing some evidence of the specificity of the association in agreement with the postulated mechanisms.^26^

### Non invasive measurements of atherosclerosis:

A number of studies have investigated the relationship between structural [carotid intima media thickness (IMT)] and functional measurements of vascular health and PD. The most convincing evidence regarding structural changes comes from epidemiological studies showing a significant cross-sectional association between periodontitis and carotid IMT in multivariate analyses.^29^–^30^ One of these two studies however failed to find a significant association between indices of PD and coronary calcium scores in a small subgroup (n=269) of the same population study.^31^

Functional measurements of vascular health have been the focus of several studies, in general small clinical samples, all have shown a significant positive association between PD and vascular health (i.e. brachial reactivity).^32^–^34^

## The role of smoking:

Smoking is an important risk factor for both PD and CVD. The strong association between smoking and these two conditions has raised concern over the true nature of the observed association between PD and CVD outcomes. Some, in fact had argued that because of the strong co-linearity among these three variables it is basically impossible to address the independent nature of the relationship between PD and CVD through multivariate adjustment (i.e. residual confounding).^35^ This important concern has been addressed by recent studies. In particular, a longitudinal investigation on the link between PD and mortality in a large sample of native American Indians with type 2 diabetes (with low prevalence of smoking and no difference in smoking between individuals with and without PD) showed a significant association between PD and cardio renal deaths.^36^ In addition a recent a longitudinal investigation, linking serum immunoglobulin G antibodies against oral bacteria to carotid IMT,^37^ and a population based case-control study linking periodontal pockets to incident acute MI found the association to be present both in smokers and never smokers.^17^ And finally in a population-based prospective study of more than 800 incident myocardial infarction survivors, PD, measured as clinical attachment loss, was associated with an approximately 40% increased risk of recurrent CVD events (fatal, non-fatal CVD or cardiac revascularizations) in patients who never smoked cigarettes.^22^ These data indicate that the association between PD and CVD cannot be the result of confounding by smoking.

## Intervention Studies:

No studies to date have been published investigating the role of periodontal treatment on clinical outcomes.

Several intervention studies have been conducted to investigate the effect of various periodontal treatments on either biomarkers of inflammation and vascular health.

## Periodontal treatment and Inflammation markers:

The studies with a randomized design and using a control group do not demonstrate consistent effects of periodontal intervention on inflammatory markers, in particular CRP.^38^–^40^ A recent report from a large multicenter pilot study to investigate the feasibility of a secondary prevention trail of PD treatment in cardiovascular disease patients confirmed the overall lack of an effect of PD treatment on CRP and suggested that the potential effect of PD treatment on CRP and other markers of inflammation could be mediated by obesity.^41^ These findings raise doubts on the importance of CRP in mediating the observed relationship between PD and CVD.

## Periodontal treatment and vascular health:

Few studies have been conducted to ascertain the effect of PD on a number of indicators of functional vascular health, these includes three small clinical studies (without placebo group) and a randomized controlled trial.^34^,^42^,^43^ All studies show consistent findings of an improvement in endothelial function (flow mediated brachial dilation) after PD treatment The randomized controlled trial^43^ showed that the effects on vascular health was dose-dependent (i.e. correlation between the clinical effects on PD and the improvement in vascular health). In addition the study showed a possible transient worsening of the vascular health following PD treatment, possibly as a result of a sudden increase in the circulatory level of the inflammatory biomarkers secondary to the mechanical removal of the periodontal plaque. Finally a recent small non controlled intervention study showed evidence for a potential benefit of PD treatment on structural markers of vascular health (IMT).^44^

## Summary:

A potential etiological link between PD and CVD may have important public health implications as both the exposure (PD) and the outcomes (CVD) are highly prevalent in industrialized societies. In situations in which both the exposure and the outcome are highly prevalent even modest associations, like those observed in the studies reporting on the link between PD and CVD outcomes, may have relevance.

If we consider the criteria for establishing the cause-effect relationship of an observed association, we can conclude that the observed association between PD and CVD outcomes satisfies most of these criteria. In particular, studies (individual and systematic reviews) to date have shown the association to be *significant*, *consistent* across different study designs and settings, *specific* to the hypothesized outcome based on postulated mechanisms, longitudinal studies have confirmed the *temporal relationship* between exposure and outcomes, elegant mechanisms have been hypothesized and confirmed to show the *plausibility* of the association that is *coherent* with the results of the laboratory evidence. Unfortunately we are missing, what is considered by many the strongest of the criteria i.e. definite evidence that the outcome can be altered (prevented or treated) by intervening on the exposure. As previously indicated, we have no definite data on the effect of periodontal treatment on CVD clinical outcomes (either in primary or secondary prevention) however it should be pointed out that the limited (both in terms of numbers and study design) experimental evidence in humans suggests a possible beneficial effect of periodontal treatment of indices of functional and structural vascular health. These same studies however have raised concerns regarding the role of inflammation and its markers as an important link between PD and CVD; in particular, the role of CRP has been called to question by the available experimental evidence.

## Conclusions:

The link between PD and CVD is worth investigating because of its potential public health implications, the evidence to data has been shown to fulfill most of the criteria established for determining a cause-effect relationship. The observed association appears to be independent from the potential confounding role of important covariates (especially smoking); studies need to be conducted to better understand the pathophysiological links between PD and CVD and this improved knowledge regarding the pathways should guide the design of specific intervention studies aimed at providing definite proof that we can, through periodontal treatment, affect CVD clinical outcomes.
